# Efficacy and safety of glucocorticoids in the treatment of COVID-19: a systematic review and meta-analysis of RCTs

**DOI:** 10.1007/s10238-024-01405-0

**Published:** 2024-07-13

**Authors:** Xiangrong Ye, Ye Li, Feng Luo, Zhibin Xu, Kaidirina Kasimu, Juan Wang, Peihang Xu, Chunjiang Tan, Hui Yi, Yifeng Luo

**Affiliations:** 1 Division of Pulmonary and Critical Care Medicine, The First Affiliated Hospital of Sun Yat-sen University, No. 58, Zhongshan Road 2, Guangzhou, 510080 Guangdong China; 2 Institute of Respiratory Diseases of Sun Yat-sen University, No. 58, Zhongshan Road 2, Guangzhou, 510080 Guangdong China; 3 Department of Organ Transplantation, The First Affiliated Hospital of Guangzhou Medical University, Guangzhou, China; 4 State Key Laboratory of Respiratory Disease, National Clinical Research Center for Respiratory Disease, Guangzhou Institute of Respiratory Health, The First Affiliated Hospital of Guangzhou Medical University, Guangzhou, China

**Keywords:** COVID-19, Corticosteroids, Clinical improvement, Mortality, Meta-analysis

## Abstract

In the realm of acute respiratory infections, coronavirus disease-19 (COVID-19), caused by severe acute respiratory syndrome coronavirus 2 (SARS-CoV-2), poses a global public health challenge. The application of corticosteroids (CSs) in COVID-19 remains a contentious topic among researchers. Accordingly, our team performed a comprehensive meta-analysis of randomized controlled trials (RCTs) to meticulously evaluate the safety and efficacy of CSs in hospitalized COVID-19 patients. To explore efficacy of CSs in the treatment of COVID-19 patients, we meticulously screened RCTs across key databases, including *PubMed, Web of Science, Embase, Cochrane Library, ClinicalTrials.gov*, as well as China's *CNKI* and *Wanfang Data*. We focused on assessing the 28 days mortality rates. We evaluated the data heterogeneity using the Chi-square test and *I*^2^ values, setting significance at 0.1 and 50%. Data from 21 RCTs involving 5721 participants were analyzed. The analysis did not demonstrate a significant association between CSs intervention and the 28 days mortality risk in hospitalized COVID-19 patients (relative risk [RR] = 0.93; 95% confidence interval [95% CI]: 0.84–1.03; *P* = 0.15). However, subgroup analysis revealed a significant reduction in 28 days mortality among patients with moderate-to-severe COVID-19 (RR at 0.85; 95% CI: 0.76–0.95; *P* = 0.004). Specifically, short-term CS administration (≤ 3 days) was associated with a substantial improvement in clinical outcomes (RR = 0.24; 95% CI: 0.09–0.63; *P* = 0.004), as was longer-term use (≥ 8 days) (RR = 0.88; 95% CI: 0.77–0.99; *P* = 0.04). Additionally, in patients with moderate-to-severe COVID-19, the administration of dexamethasone increased the number of 28 days ventilator-free days (Mean Difference = 1.92; 95% CI: 0.44–3.40; *P* = 0.01). Methylprednisolone also demonstrated significant benefits in improving clinical outcomes (RR = 0.24; 95% CI: 0.09–0.63; *P* = 0.004). Our meta-analysis demonstrated that although there is no significant difference in 28 days mortality rates among hospitalized COVID-19 patients, the use of CSs may be beneficial in improving clinical outcomes in moderate or severe COVID-19 patients. There was no significant increase in the occurrence of adverse events associated with the use of CSs. Our meta-analysis provides evidence that while CSs may not be suitable for all COVID-19 patients, they could be effective and safe in severely ill COVID-19 patients. Consequently, it is recommended to administer CSs for personalized treatments in COVID-19 cases to improve the clinical outcomes while minimizing adverse events.

**Supplementary Information:**

The online version contains supplementary material available at 10.1007/s10238-024-01405-0.

## Introduction

The COVID-19, caused by SARS-CoV-2, has become the most severe global public health emergency and greatly impacted the world since its outbreak at the end of 2019 [1]. The COVID-19 infection is characterized by increased mobility and high mortality rate, especially among the elderly patients with underlying diseases [2]. By December 6, 2023, this pandemic has resulted in over 772 million cases and 6.98 million fatalities worldwide, presenting unprecedented challenges to human health and healthcare systems [3].

For individuals with confirmed COVID-19, novel targeted therapeutics for COVID-19 treatment still remain limited. To date, the supportive care remains the primary medical interventions for COVID-19 cases, and medications often prove ineffective and may carry the risk of toxicity, particularly when used in combination [4]. It is noteworthy that the World Health Organization recommended the application of CSs in severe and critical COVID-19 patients [5]. Initial recommendations from the Infectious Diseases Society of America in September 2022 were against the routine use of CSs for COVID-19, unless there were critically ill COVID-19 hospitalized patients or other reasons, such as asthma, or refractory shock [6]. However, the efficacy and safety of CSs in COVID-19 patients have been a debate within the medical community [7]. On the one hand, several studies underscore the potential therapeutic benefits of CSs administration; on the other hand, there is a growing body of literature highlighting adverse outcomes associated with CSs. For example, Li and colleagues found the use of CSs in COVID-19 patients is associated with prolonged viral RNA shedding, raising concerns about potential adverse effects [8, 9]. In addition, there were several studies that have shown either no benefit or an increased mortality in different subgroup of cases [10–13]. This ongoing controversy highlights the critical need for continued and comprehensive systematic review and meta-analysis to provide more evidence for the efficacy of CSs therapy in COVID-19 cases.

Considering all factors mentioned above, we conducted a systematic review and meta-analysis to assess the safety and efficacy of CSs therapy in hospitalized COVID-19 patients, juxtaposed with non-steroid treated groups and cohorts receiving different steroid dosages. Moreover, our analysis further analyzed different CSs subgroups in terms of disease severity, dosage, the specific type of CSs employed, and the duration of treatment. Our aim is to offer more therapeutic options to physicians and the medical community.

## Methods

### Databases and search strategy

In accordance with the rigorous standards set by the PRISMA declaration, our study was duly registered on the PROSPERO platform (CRD42023486275). Our methodological approach entailed an extensive and strategic literature search across a suite of databases including PubMed, Web of Science, Embase, Cochrane Library, ClinicalTrials.gov, CNKI, and Wanfang Data. We designed a comprehensive search algorithm, blending both MeSH terms and free-text terms in a Boolean framework. The MeSH terms are listed as follows: COVID-19, glucocorticoid, dexamethasone, prednisolone, and prednisone. The free-text terms are SARS-CoV-2, 2019 Novel Coronavirus, methylfluorprednisolone, hexadecadrol, and so on (see Table 1 of Supplemental Material file online for more details regarding the search strategies). The search string was crafted to capture a wide range of literature related to COVID-19 and CSs therapy, leading to a comprehensive retrieval of 5757 articles, including one identified through manual search, up to the cutoff date of November 21, 2023.

### Eligibility criteria

This investigation entailed a systematic appraisal of RCTs, following stringent criteria encompassing participant characteristics, intervention strategies, comparison groups, outcome measures, and study designs. Inclusion was limited to patients aged 18 and above, diagnosed with COVID-19 according to established clinical guidelines [14]. The interventions under review were various CSs therapies, set against control groups receiving either alternate dosages of CSs, standard supportive care, or placebo [14].

Study selection was undertaken independently by two researchers (Xiangrong Ye and Ye Li), who diligently reviewed titles and abstracts for relevance. Disparities in selection were resolved through joint discussions or by consulting a third arbitrator (Feng Luo). For articles lacking complete data, efforts were made to obtain additional information from the study's corresponding authors. The Cochrane Risk of Bias Tool 2.0 was utilized to assess the methodological robustness and potential bias of the studies included [15].

### Data collection process

The primary outcomes of this investigation were the 28 days mortality rate. The secondary outcomes evaluated included the duration of hospitalization, the number of days free from mechanical ventilation within the first 28 days, rates of ICU transfer, and the 60 days mortality rate. The study also analyzed adverse events, specifically focusing on secondary infections such as bacteremia and fungal infections, as well as significant adverse events categorized as Grade 3–4. Patients requiring prolonged CSs or immunosuppressive treatment for chronic ailments were systematically excluded. Data collation and extraction were executed independently by two distinct groups, ensuring the integrity and precision of the data collection process.

### Assessment of risk of bias and quality of evidence

In conducting this study, evaluators Ye and Li independently applied the Cochrane Risk of Bias Tool for a detailed and systematic assessment of the randomized controlled trials under review. This comprehensive evaluation targeted seven critical aspects: (1) the methodology of random sequence generation, (2) the integrity of allocation concealment, (3) the effectiveness of blinding for participants and personnel, (4) the impartiality of outcome assessment blinding, (5) the completeness and reliability of outcome data, (6) the presence of selective reporting biases, and (7) the potential influence of other bias sources. Each domain was meticulously appraised, classified into categories of 'low,' 'unclear,' or 'high' risk of bias, with the overarching bias risk for each trial being determined by the highest risk level noted across these domains.

### Subgroup analysis

Subgroup analyses within the study focused on several pivotal variables: CSs type (dexamethasone, methylprednisolone, and placebo), patient condition severity (mild, moderate to severe), duration of treatment (≤ 3 days, 4–7 days, ≥ 8 days), and dosage intensity (high at > 2 mg/kg/day, low at ≤ 2 mg/kg/day).

### Data analysis

For the statistical analysis, the Review Manager (RevMan) version 5.3 was used, a tool by the Cochrane Collaboration. Heterogeneity among study results was assessed with Chi-square and *I*^2^ tests, applying thresholds of 0.1 and 50%, respectively, under a random-effects model framework. Preference was given to the *I*^2^ test results in cases of inconsistency. The Mantel–Haenszel method was utilized for binary data synthesis, deriving pooled RR along with their 95% CIs. Continuous outcomes were analyzed using the inverse variance approach, with mean differences (MDs) and corresponding 95% CIs as the metrics. In situations where only medians and interquartile ranges were reported, the mean–variance estimation method was employed for mean and standard deviation estimations [16]. Publication bias was probed using the funnel plots, Egger’s test, and Begg’s test [17].

## Results

### Study selection and study characteristics

**Fig. 1:**
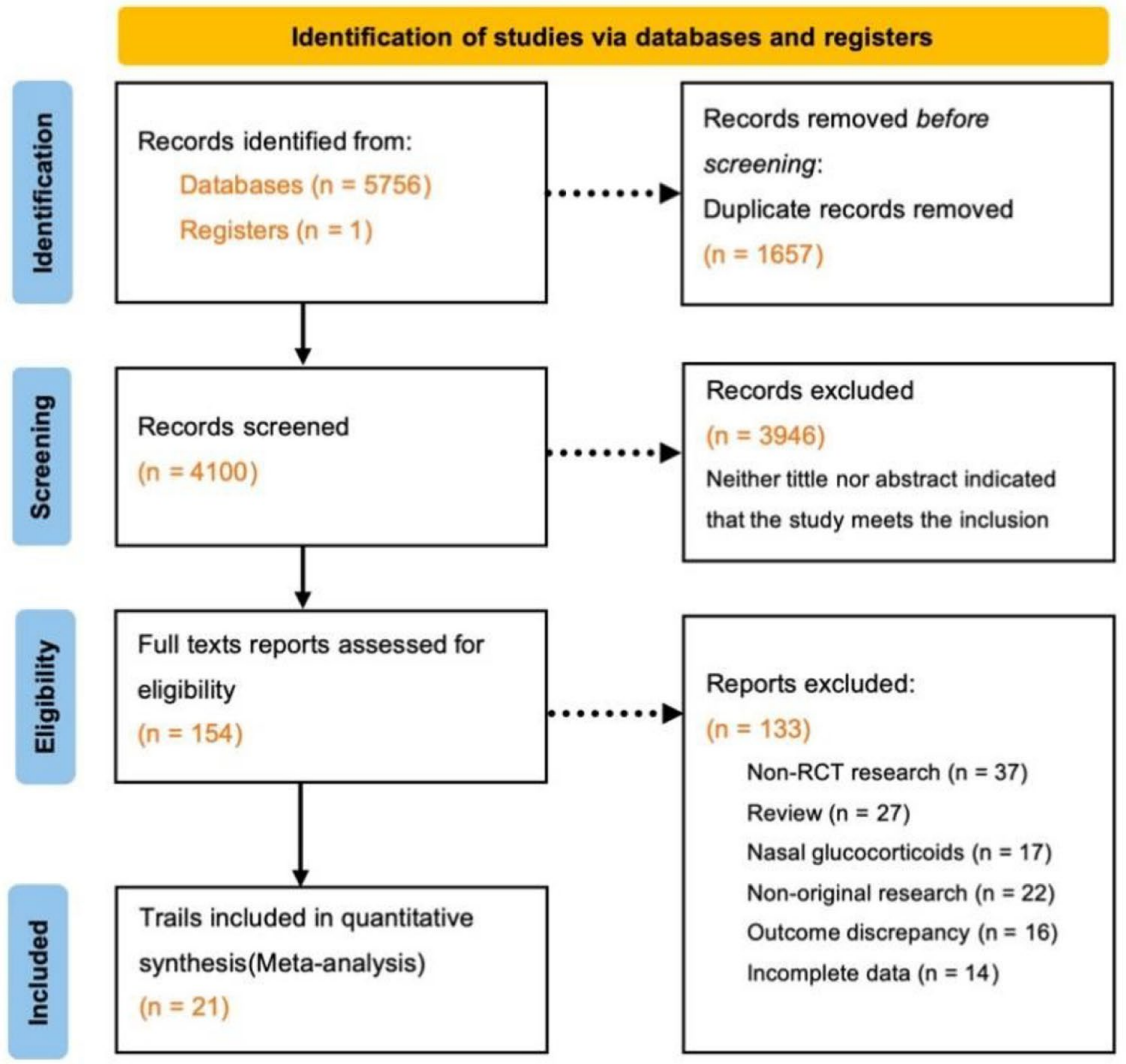
PRISMA flow diagram for search

Utilizing the defined PICOS criteria, this systematic review initially retrieved a total of 5757 publications. After removing 1657 duplicates and subsequent excluding 3946 records based on title and abstract assessments, 154 articles were subjected to full-text evaluation. This process culminated in the inclusion of 21 studies for detailed data extraction and analysis, as depicted in Fig. 1, Tables 1, and 2. The flow diagram was created in accordance with PRISMA guidelines [18]. Published between 2020 and 2023, these studies collectively involved 5721 participants and were all RCTs. Among them, 15 studies [19–33] compared CSs therapy against standard treatment, three [34–36] used a placebo as the comparator, and three [37–39] utilized low-dose CSs. Methylprednisolone was the intervention in nine studies [19, 21, 22, 25, 29, 30, 34–36], while dexamethasone was used in twelve [20, 23, 24, 26–28, 31–33, 37–39], with treatment durations ranging from 3 to 10 days.

**Table 1:** Main characteristics of the included studies

Author	Study site	Design	Sample size	Dosage and administration		Outcomes reported
				Intervention	Control	
Maryam [19]	Iran	Randomized controlled clinical trial	34 intervention, 28 control	MP pulse IV 250 mg/day for 3 days	Standard care	Mortality at 28 days, time to event (discharge or death) days and adverse events
Somayeh [37]	Iran	Randomized clinical trial	58 intervention, 61 control	DM high-dosage IV 24 mg/day for 3 days, then 8 mg/day for the at least 4 days	DM low dosage IV 8 mg/day for at least 7 days	Mortality at 28 days, duration of hospitalization, ICU admission and adverse events
Bruno [20]	Brazil	Randomized clinical trial	151 intervention, 148 control	DM IV 20 mg/day for 5 days, 10 mg/day for 5 days	Standard care	28-VFD, mortality at 28 days, SOFA score and adverse events
Cameron [38]	USA	Randomized, double-blind, controlled trial	494 intervention, 516 control	DM IV 6 mg/day for up to 10 days	Baricitinib orally 4 mg/day for up to 14 days	mortality at 28 days and adverse events
Abbas [21]	Iran	Prospective three-arm randomized clinical trial	36interventionA, 35 interventionB, 35 control	MP IV 16 mg twice a day for up to 10 days	Standard care	Need for mechanical ventilation, 28-VFD, duration of hospitalization, ICU stay days, mortality at 28 days and adverse events
Francesco [22]	Italy	Randomized controlled trial	337 intervention, 340 control	MP IV 80 mg/day for 8 days	Standard care	Mortality at 28/60 days, duration of hospitalization, ICU admission and adverse events
Marie [23]	Denmark	Randomized clinical trial	503 intervention, 497 control	DM high-dosage IV 2 mg/day for 10 days	Standard care	The median number of days alive without life support, mortality at 28/90 days and adverse events
Carlo [36]	Italy	Randomized,double-blind,placebo-controlled trial	151 intervention, 150 control	MP IV 1 g/day for 3 days	Placebo	Discharge without oxygen, mortality at 28 days, ICU admission and adverse events
Carlos [24]	USA	Randomized, open-label, clinical Trial	70 intervention, 72 control	DM high-dosage IV 0.2 mg/kg/day with a maximum dose of 20 mg for 10 days	Standard care	Mortality at 28 days, ICU admission, ICU stay days and adverse events
Corral [25]	Spain	Randomized, open-label, clinical Trial	63 intervention, 62 control	MP IV 6 mg/day for 10 days	Standard care	Mortality at 28 days, ICU admission, MV duration, duration of hospitalization and adverse events
Hamidreza [26]	Iran	Randomized clinical trial	25 intervention, 25 control	DM IV 20 mg/day for 5 days and then at 10 mg/day for 5 days	Standard care	Mortality at 28 days, duration of hospitalization, ICU stay days
Lila [27]	France	Randomized clinical trial	270 intervention, 276 control	DM high-dosage IV 20 mg/day for 5 days and then at 10 mg/day for 5 days	Standard care	Overall survival at 60 days, viral load evolution, 28-VFD, duration of hospitalization and adverse events
Xiao [34]	China	Single-blind, randomized, control trial	43 intervention, 43 control	MP IV 1 mg/kg/day for 7 days	Placebo	Clinical deterioration 14 days after randomization, ICU admission, mortality at 28 days and duration of hospitalization
Manuel [28]	Spain	Randomized, open-label, clinical trial	98 intervention, 102 control	DM high-dosage IV 20 mg/day for 5 days, and then 10 mg/day for 5 days	Standard care	Clinical worsening within 11 days, mortality at 28 days, MV duration and adverse events
Luis [29]	Spain	Randomized, open-label, clinical trial	35 intervention, 29 control	MP IV 40 mg bid for 3 days and then 20 mg bid for 3 days	Standard care	Composite endpoint (ICU admission, need of NIV or death), duration of hospitalization and adverse events
Christiane [35]	Brazil	Randomized, double-blind, phase IIb, placebo-control trial	194 intervention, 199 control	MP IV 0.5 mg/kg twice daily for 5 days	Placebo	Mortality at 7/14/28 days, virological clearance at day 5/7 and duration of hospitalization
Raef [30]	USA	a single pretest, single posttest quasi-experiment	132 intervention, 81 control	MP IV 0.5 to 1 mg/kg/day divided in 2 doses for 3 days	Standard care	Mortality at 28 days, MV duration, duration of hospitalization and adverse events
Naveen [31]	IND	Randomized controlled trial	21 intervention, 21 control	DM high-dosage IV 20 mg/g for 3 days plus standard care*	Tocilizumab, 6 mg/kg plus standard care*	28-VFD, MV duration, mortality at 28 days, duration of hospitalization and adverse events
Luis [32]	Argentina	Randomized, open-label, clinical trial	49 intervention, 49 control	DM high-dosage IV 16 mg/day for 5 days and then 8 mg/day for 5 days	Standard care	28-VFD, MV duration, mortality at 28 days, duration of hospitalization and adverse events
Montalvan [39]	Italy	Randomized non-blinded, control trial	41 intervention, 40 control	DM high-dosage IV 24 mg/day	DM low dosage IV 24 mg/day	Mortality at 28 days, duration of hospitalization and adverse events
Huimin [33]	USA	Randomized clinical trial	52 intervention, 55 control	DM high-dosage IV 20 mg/day for 5 days, and then 10 mg/day for 5 days	Standard care	Clinical improvement at day 28, mortality at 28 days, ICU-free days, 28-VFD, and adverse events

Abbreviations: MP: methylprednisolone; DM: dexamethasone; MV: mechanical ventilation; VFD: ventilator-free days; ICU: intensive care unit; SOFA, Sequential Organ Failure Assessment; Standard care: dexamethasone 6 mg for 10 days; IV: intravenous injection

**Table 2:** Reported outcomes in the included studies

Author	Population	Mean age (% female)	Information collection	OR/RR/HR	95%CI	Outcomes
Maryam [19]	Hospitalized severe COVID-19 patients	58.5 (37.1%)	Excel database	0.293	0.154–0.556	Methylprednisolone pulse could be an efficient therapeutic agent for hospitalized severe COVID-19 patients at the pulmonary phase
Somayeh [37]	Hospitalized COVID-19 patients	60.77* 57.28** (50.4%)	Medical records	–	–	Low- versus high-dose dexamethasone therapy did not affect the outcomes
Bruno [20]	Moderate or severe ARDS COVID-19 patients	61 (37.5%)	Medical records	0.97	0.72–1.31	Intravenous dexamethasone resulted in a statistically significant increase in the number of ventilator-free days over 28 days
Cameron [38]	Hospitalized adults with COVID-19 who required supplemental oxygen	58.3 (41.6%)	Dataset	1.21	0.72–2.04	Both resulted in similar mechanical 28-VFD, but dexamethasone was associated with significantly more adverse events
Abbas [21]	Mild or moderate ARDS COVID-19 patients	63.05* 62.8** (40.8%)	Standardized data collection form	–	–	Severe COVID-19 treated with dexamethasone might have a better clinical status at 28-day follow-up compared to methylprednisolone at an equivalent dose
Francesco [22]	Hospitalized adults with COVID-19 who required supplemental oxygen	64.4* 63** (30.6%)	Electronic case report forms	1.04#	0.54–2.00	Prolonged, higher-dose methylprednisolone did not reduce mortality at 28 days compared with conventional dexamethasone in COVID-19 pneumonia
Marie [23]	Patients with COVID-19 and severe hypoxemia	65 (30.5%)	Web-based case report forms	0.86&	–	12 mg/day of dexamethasone compared with 6 mg/day of dexamethasone did not result in statistically significantly more days alive without life support at 28 days
Carlo [36]	Patients hospitalized with COVID-19 pneumonia	64 (27.9%)	Clinical database	0.92$	0.71–1.20	Methylprenisolone pulse therapy added to dexamethasone was not of benefit in patients with COVID-19 pneumonia
Carlos [24]	Patients with COVID-19 and hypoxemia	55.46* 57.22** (30.3%)	REDCap electronic data capture tools	–	–	The use of weight-based dexamethasone dosing was not superior to dexamethasone 6 mg in reducing all-cause mortality at 28 days
Corral [25]	Hospitalized adults with COVID-19 who required supplemental oxygen	60 (34.0%)	Dataset	–	–	250 mg/day for 3 days of methylprednisolone compared with 6 mg/day for 10 days of dexamethasone did not result in a decrease in mortality or intubation
Hamidreza [26]	Mild-to-moderate ARDS COVID-19 patients	62.28 (28.0%)	Dataset	–	–	Corticosteroid administration had no clinical benefit in patients with COVID-19-induced mild-to-moderate ARDS
Lila [27] France	ICU-admitted adults with COVID-19 AHRF	67.4 (24.2%)	Dataset	0.96##	0.69–1.33	High-dose dexamethasone did not significantly improve 60 days survival
Xiao [34]	Adult patients with COVID-19 pneumonia	56 (52.3%)	Dataset	0.977***	0.933–1.023	Short-term early use of corticosteroid could suppress the immune cells, which may prolong severe ARDS patients with COVID-19 pneumonia
Manuel [28]	Patients with COVID-19 who required supplemental oxygen	64 (38.0%)	Dataset	1.129&	0.338–3.772	High dose of dexamethasone reduced clinical worsening within 11 days after randomization, compared with low dose
Luis [29] Spain	Patients with COVID-19 who required supplemental oxygen	70 (39.1%)	Database	–	–	The use of methylprednisolone had no significant effect on the primary endpoint in ITT analysis
Christiane [35]	Hospitalized patients who had clinical or radiological suspicion of COVID-19	55 (35.4%)	Electronic database (REDCap)	0.924&	0.669–1.275	A short course of methylprednisolone in hospitalized patients with COVID-19 did not reduce mortality in the overall population
Raef [30]	Moderate or Severe ARDS COVID-19 patients	62 (48.8%)	Standardized electronic case report form	–	–	An early short course of methylprednisolone reduced escalation of care and improved clinical outcomes
Naveen [31]	Moderate or Severe ARDS COVID-19 patients	50.5 (42.9%)	Dataset	–	–	HDD was associated with a very high rate of mortality and adverse events
Luis [32] Argentina	Patients with confirmed COVID-19-related ARDS	61.8 (29.6%)	Dataset	1.6&	1.1–2.33	Use of higher doses of dexamethasone compared with the recommended low-dose treatment did not show an increase in VFD
Montalvan [39]	Patients hospitalized with COVID-19 pneumonia	57.2 (43.2%)	Dataset	1.95^	1.1–3.5	High dose of dexamethasone was associated with increased mortality, risk of intubation and nosocomial infections
Huimin [33]	Hospitalized patients with COVID-19 pneumonia	57.0 (47.7%)	Dataset	1.45$	0.55–3.86	Dexamethasone 20 mg daily did not result in better clinical outcome improvement and was probably associated with higher 28 days mortality

Abbreviations: IV: intravenous injection;*: intervention;**:control;#: noninvasive ventilation; &: motality of 28 days; $: median time to discharge/improvement; AHRF: acute hypoxemic respiratory failure; ##: 60 days mortality; ***: in-hospital mortality; ^: becomes critically ill and requires intubation

### Risk of bias and quality of evidence

**Fig. 2:**
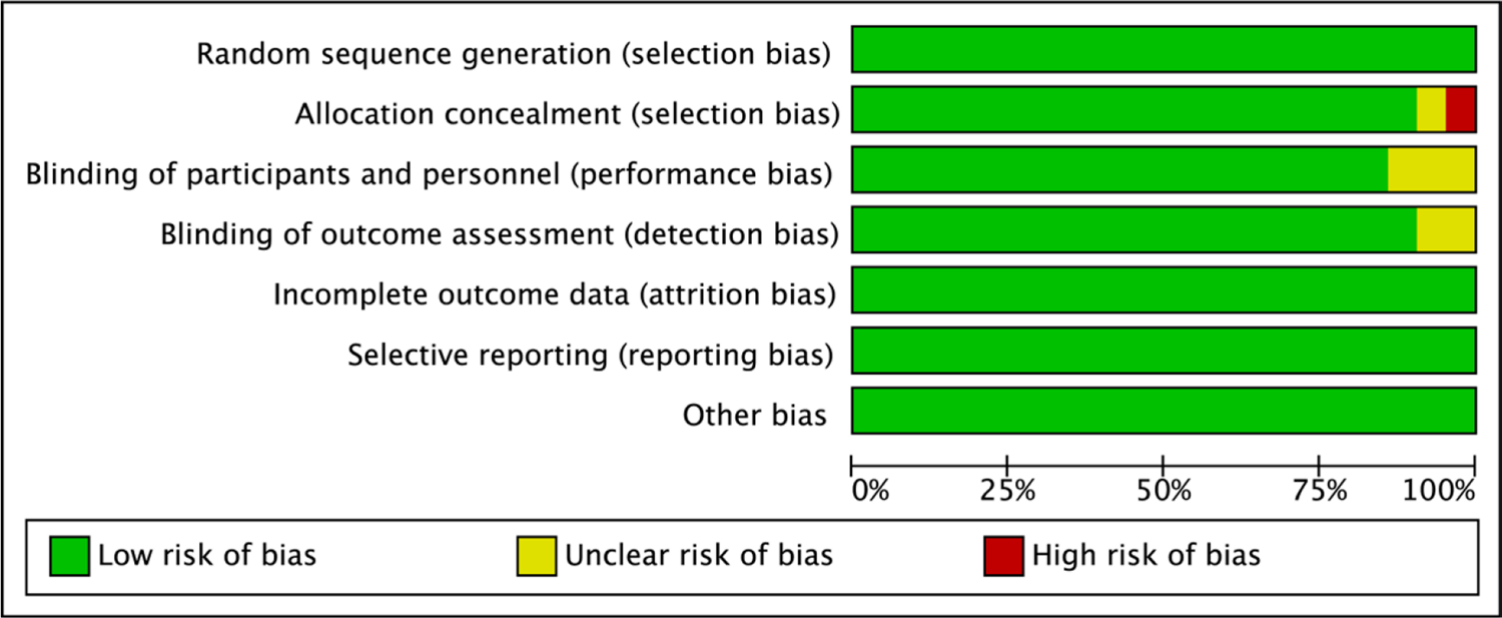
Risk of bias graph

**Fig. 3:**
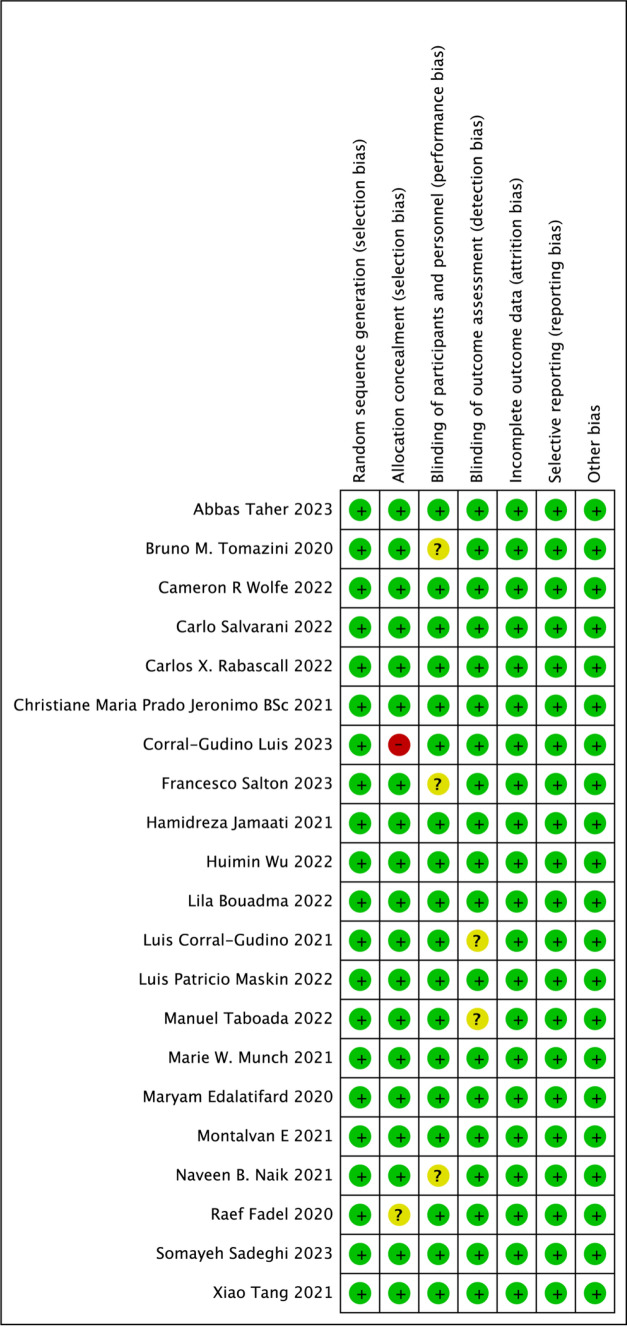
Risk of bias summary

Within the analyzed cohort of studies, fourteen were adjudged to have a low risk of bias. Six studies were categorized as having unclear risk, primarily due to ambiguities in the implementation of blinding, measurement of outcomes, and descriptions of allocation concealment. One study was identified to present a high risk of bias, specifically attributed to inadequacies in allocation concealment (Figs. 2 and 3).

### Primary outcomes: 28 days mortality

**Fig. 4:**
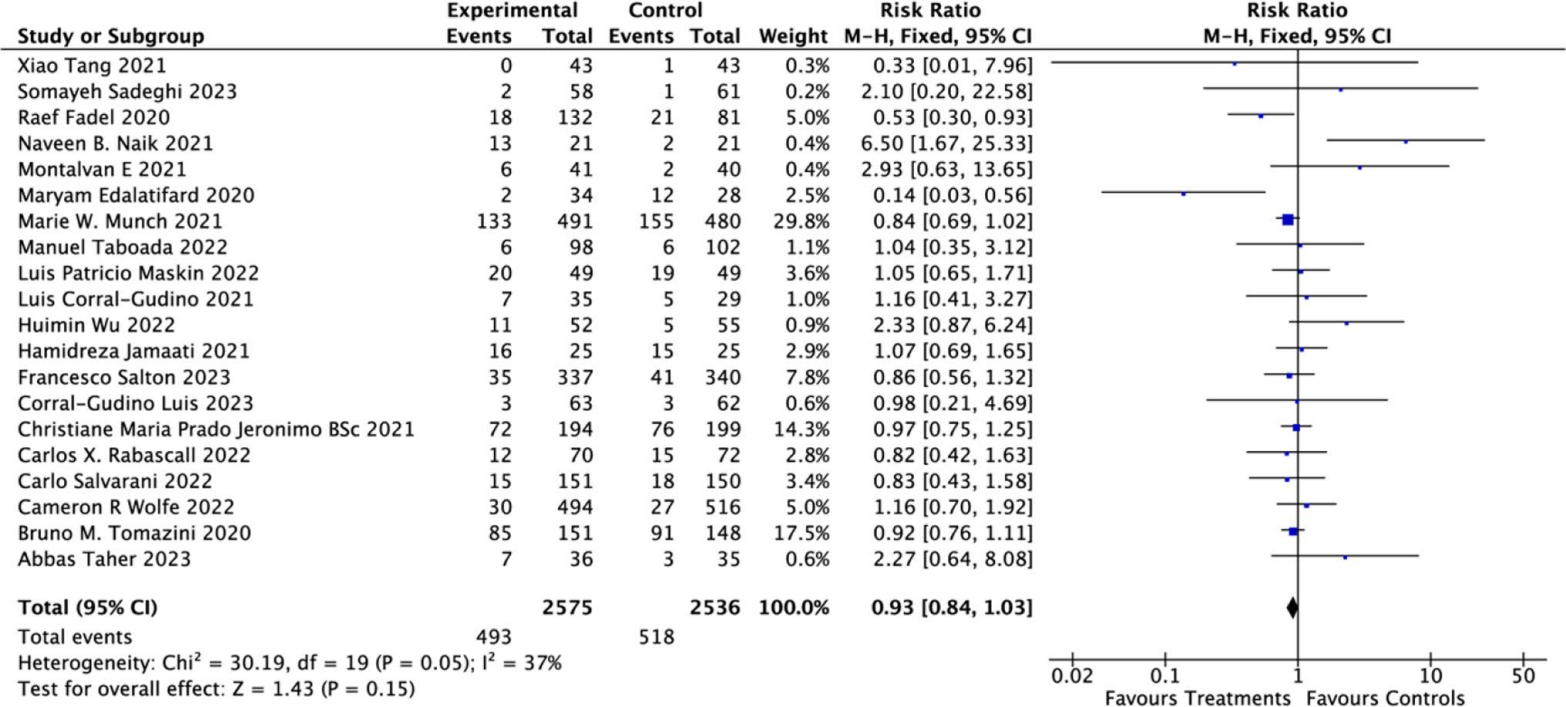
Effect of corticosteroids on mortality at 28 days among hospitalized patients

Across 20 studies [19, 23, 28–32, 34, 35, 37, 39], involving 4898 subjects, the 28 days mortality rates were analyzed. Within the CSs-treated cohort, the mortality rate stood at 19.4%, whereas the control group exhibited a rate of 20.2%. The computed RR was 0.93 (95% CI: 0.84–1.03; *P* = 0.15), suggesting there is no statistically significant correlation between CSs intervention and the control group with regard to the 28 days mortality rate among hospitalized COVID-19 patients (Fig. 4). Thus, CSs treatment did not confer a reduced risk of 28 days mortality. The heterogeneity assessment, conducted using Chi-square and *I*^2^ tests, indicated the absence of significant heterogeneity (30.19 and 37%, respectively).

### Secondary outcomes

**Fig. 5:**
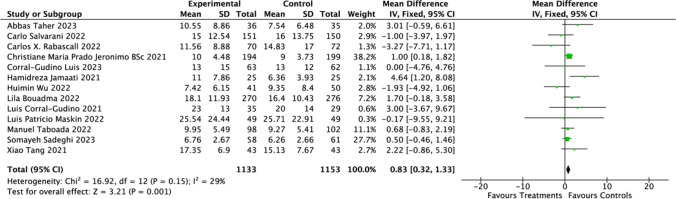
Effect of corticosteroids on hospitalization durations among hospitalized patients

**Fig. 6:**
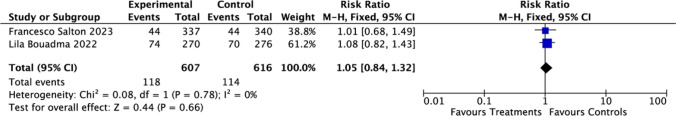
Effect of corticosteroids on mortality at 60 days among hospitalized patients

**Fig. 7:**
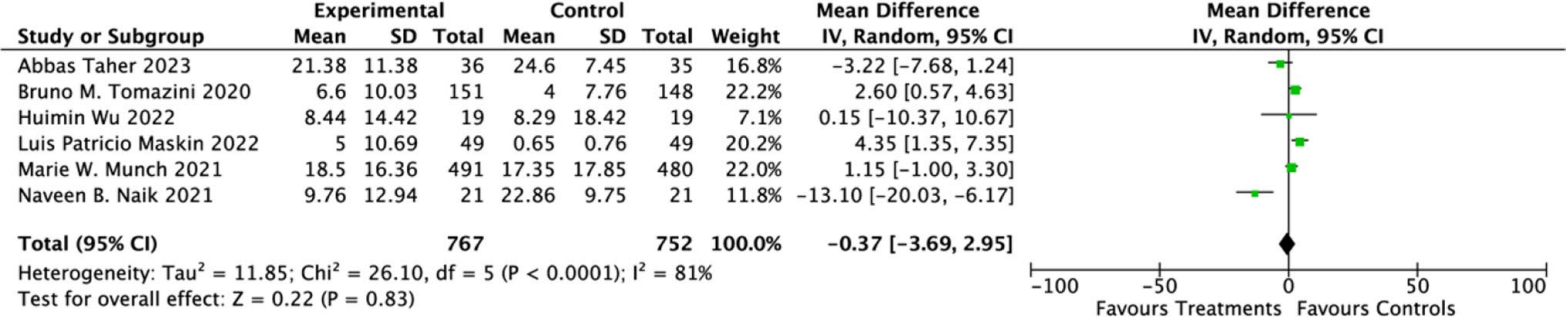
Effect of corticosteroids on 28 days ventilator-free days among hospitalized patients

Analyzing data from 13 studies [21, 24–29, 32–37] focused on the impact of CSs treatment on hospitalization durations in COVID-19 patients, we found a mean difference (MD) of 0.83 days (95% CI: 0.32 to 1.33; *P* = 0.001). This indicates the use of CSs is associated with longer hospital stays (Fig. 5). Regarding 60 days mortality rates, the results (RR = 1.05; 95% CI: 0.84 to 1.32; *P* = 0.66) (Fig. 6) indicated no significant association with CSs use. Contrastingly, a significant reduction in 28 days ventilator-free days was observed in hospitalized COVID-19 patients (RR = 0.83; 95% CI: −3.69 to 2.95; *P* = 0.83) (Fig. 7). Moreover, the investigation revealed marked heterogeneity in the reports on hospital stay duration (Chi-square = 16.92, *I*^2^ = 29%) and 28 days Ventilator-Free Days (Chi-square = 26.10, *I*^2^ = 81%), but not in the 60 days mortality data (Chi-square = 0.08, *I*^2^ = 0%).

### Adverse events

**Fig. 8:**
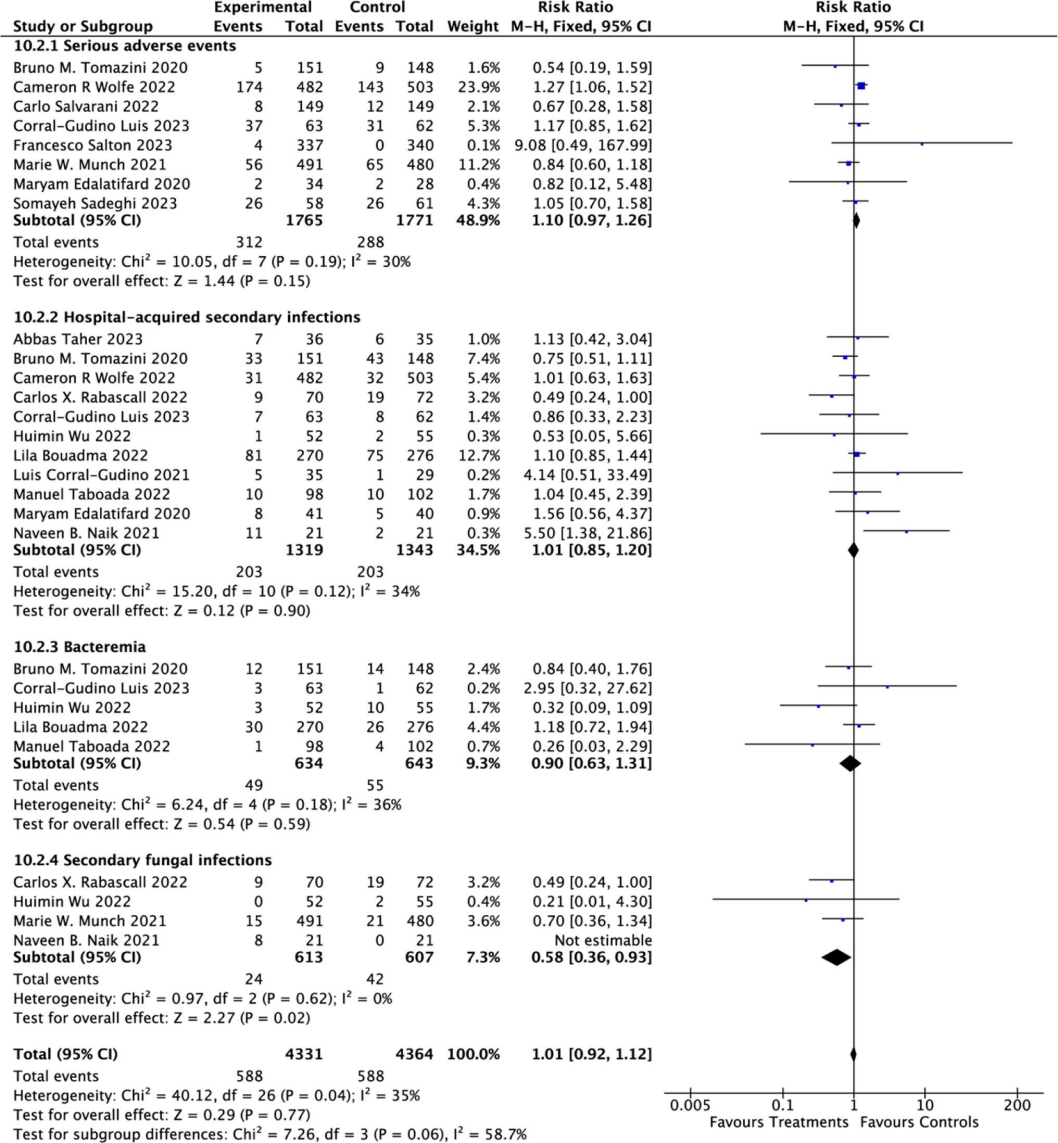
Adverse events of corticosteroids among hospitalized patients

Our analysis revealed no significant differences in the incidence of serious adverse events among hospitalized COVID-19 patients [19, 20, 22, 23, 25, 36–38] (RR = 1.10; 95% CI: 0.97 to 1.26; *P* = 0.15), hospital-acquired secondary infections [19–21, 24, 25, 27–29, 31, 33, 38] (RR = 1.01; 95% CI: 0.85 to 1.20; *P* = 0.90), bacteremia [20, 25, 27, 28, 33] (RR = 0.90; 95% CI: 0.63 to 1.31; *P* = 0.59), and secondary fungal infections [23, 24, 31, 33] (RR = 0.58; 95% CI: 0.36 to 0.93; *P* = 0.02). Statistical analyses demonstrated no meaningful heterogeneity in studies examining serious adverse events (Chi-square = 10.05, *I*^2^ = 30%), nosocomial infections (Chi-square = 15.20,* I*^2^ = 34%), and bacteremia (Chi-square = 6.24, *I*^2^ = 36%). In COVID-19 patients, high-dose CSs therapy significantly reduced the incidence of fungal infections, and these findings showed homogeneity in statistical analysis (Chi-square = 0.97, *I*^2^ = 0%) (Fig. 8).

### Subgroup analysis

The subgroup analyses, based on disease severity, demonstrated that CSs application in moderate or severe COVID-19 patients had lower rates of the 28 days mortality [20, 22, 24, 25, 28, 29, 35, 36, 39] and longer 28 days ventilator-free days [20, 23] compared with those without CSs application (Table 3, online supplemental Figs. 1 and 2). Subgroup analyses according to CSs type showed there is no statistically significant difference in 28 days mortality rates among patients treated with dexamethasone [23, 24, 28, 32, 33, 37, 39], methylprednisolone [19, 29, 30, 35, 36], or a combination therapy of these two CSs [21, 22, 25] (Table 3, online supplemental Fig. 3). Notably, despite significant heterogeneity (Chi-square = 10.63,* I*^2^ = 81%), the treatment of methylprednisolone alone [19, 30, 36] (RR = 0.24; 95% CI: 0.09 to 0.63; *P* = 0.004) significantly reduced the risk of 28 days mortality (RR = 0.24; 95% CI: 0.09 to 0.63; *P* = 0.004) when specifically analyzing moderate or severe COVID-19 patients (Table 3, online supplemental Fig. 4). Importantly, patients receiving short-term CSs application (≤ 3 days) [19, 30, 36] or treatments extending ≥ 8 days [20, 22–25, 28] tended to exhibit a lower rate of 28 days mortality when compared with those with an intermediate duration of CSs (4–7 days) [29, 35, 37] (Table 3, online supplemental Fig. 5). Analysis of the studies involving methylprednisolone alone and CSs usage of ≤ 3 days showed high heterogeneity, whereas most other analyses had low heterogeneity (Table 3).

**Table 3:** Subgroup analysis of the variables influencing factors with clinical outcomes of COVID-19

Subgroup factor		Clinical outcomes	RR/MD^	95%CI	*P* value	Heterogeneity Chi-square/*I*^2^
Moderate or severe patients		28 days mortality rates	0.85	0.76–0.95	0.004*	11.95/8%
		28 days ventilator-free days	1.92^	0.44–3.40	0.01*	0.92/0%
Corticosteroid type	Dexamethasone	28 days mortality rates	0.99	0.76–1.28	0.94	7.37/19%
	Methylprednisolone	28 days mortality rates	0.44	0.18–1.10	0.08	50.32/92%
	combination	28 days mortality rates	0.95	0.64–1.42	0.82	2.01/1%
Treatment duration	≤ 3 days	28 days mortality rates	0.24	0.09–0.63	0.004*	10.63/81%
	4–7 days	28 days mortality rates	0.99	0.77–1.27	0.93	0.50/0%
	≥ 8 days	28 days mortality rates	0.88	0.77–0.99	0.04*	0.57/0%

RR: relative risk; MD: mean difference; CI: confidence interval; *: have statistical significance

## Discussion

In contrast with previously published meta-analyses, our study presents several distinct advantages. It incorporates an expanded cohort of 21 RCTs, including cases of moderate or severe COVID-19. In addition, our research features a larger sample size, which enhances the quality of this study. In our findings, the benefits of CSs application were less evident in the general hospitalized patient population. Nevertheless, our findings show that CSs significantly reduce the 28 days mortality rate and increase the VFD over a 20 days period in COVID-19 patients with moderate or severe ARDS, which is possibly due to variations in disease severity and the diversity of CSs dosages administered to COVID-19 patients.

Focusing on COVID-19, the pharmacodynamics of hydrocortisone and dexamethasone and their impact on COVID-19 patients have been highlighted in recent WHO-sponsored trials and meta-analyses [40]. Our findings are in line with the RECOVERY trial, which demonstrated the reduction in 28 days mortality for patients with hypoxia or mechanical ventilation when treated with dexamethasone [41].

Based on results of our analysis, the use of CSs did not increase the risk of serious adverse events, secondary infections, bacteremia, and fungal infections in COVID-19 patients with ARDS. It is notable that, in severe COVID-19 cases, CSs application was associated with a reduced risk of fungal infections. This may be attributed to the potent anti-inflammatory effects of CSs, which can improve clinical outcomes [42]. Critically ill COVID-19 patients may benefit even more from higher dosage of CSs [43]. Furthermore, some studies suggest potential benefits of inhaled CSs in preventing disease deterioration and secondary organ damage in patients with COVID-19, which could be a new therapeutic option [44, 45].

In the present study, CSs treatment was not found to improve the disease prognosis in certain subgroups, such as the intermediate treatment duration (4–7 days) of CSs and the combination therapy of dexamethasone and methylprednisolone and intermediate duration (4–7 days) of CSs treatment, underscoring the necessity for further targeted clinical research [2]. As such, the current body of evidence does not support a universal application of CSs therapy among all hospitalized COVID-19 patients, and further stratified clinical research is required to demonstrate its efficacy [5]. This underscores the critical necessity for careful consideration in the clinical decision-making regarding the use of CSs as the clinical application of CSs is fraught with complexity. Inappropriate administration of CSs is associated with heightened risks of infection, osteoporosis, hyperglycemia, and bleeding, potentially worsening the patient's condition [46, 47].

Accordingly, identifying the optimal timing for CSs therapy is crucial. Our subgroup analysis demonstrates that durations of CSs treatment (≤ 3 days or ≥ 8 days) can significantly reduce mortality in severe COVID-19 patients, which may be related to the timing of CSs application during the inflammatory storm. An observational cohort study provides evidence that early short course of CSs combined with furosemide reduced the 28 days mortality in non-critically ill COVID-19 patients [48]. Findings from L. Mahajan and collaborators highlight that 10 days of dexamethasone treatment reduced the number of ICU admissions and mortality among COVID-19 patients [49]. Due to this result from subgroup analysis, we could emphasize there may be biased. Further clinical trials are required to confirm the conclusion from subgroup analysis.

Variations in recommended treatment durations for CSs might be attributed to the distinct metabolic pathways activated by different CSs, such as methylprednisolone and dexamethasone. They exert potent anti-inflammatory effects by binding with glucocorticoid response elements, modulating transcription factors, and influencing secondary messenger pathways [50]. This underscores the significance of selecting the appropriate CSs type for COVID-19 treatment [51], as the choice of CSs for treating COVID-19 remains an area of ongoing exploration. New clinical studies are required to explore the specific populations that may benefit from CSs, as well as to refine the timing and duration of CSs application, with the aim of optimizing the benefits of CSs while minimizing their potential harm.

### Limitations

We acknowledge several inherent limitations. Firstly, considering the methodology of systematic reviews and meta-analyses, the issue of heterogeneity is inevitable, exacerbated by the diversity in patient demographics, therapeutic approaches, and the types of CSs administered. The second limitation arises from the inadequate reporting of adverse events in certain studies we analyzed, which constrains the conclusiveness of subgroup analysis in this context. Moreover, the short follow-up periods in the included RCTs limit the understanding of long-term adverse effects of CSs administered at varying doses and durations. This necessitates further research through observational studies or additional randomized trials to thoroughly understand the prolonged implications of CSs therapy in such cases.

## Conclusion

CSs have been identified as an effective therapeutic intervention for reducing mortality risk in patients with severe COVID-19 pneumonia. Dose, duration, and underlying comorbidities of CSs treatment are key factors for clinical outcome of severe COVID-19. In our research, although there was no significant difference in 28 days mortality rates among hospitalized COVID-19 patients, our results show that CSs may be beneficial in improving clinical outcomes by choosing appropriate duration and type of application specially for COVID-19 patients with moderate or severe ARDS. Additionally, there was no significant increase in the incidence of adverse events associated with the use of CSs. It should be noted that our research has inherent limitations and bias is inevitable. Consequently, it is recommended to administer CSs for an personalized duration in moderate or severe COVID-19 cases to improve the clinical outcomes while minimizing adverse events. Our meta-analysis provides evidence that CSs are not suitable for all COVID-19 patients, but they could be effective and safe in severely ill COVID-19 patients.

## Supplementary Information

Below is the link to the electronic supplementary material.

Supplementary file1 (DOCX 11141 KB)

## Data Availability

The datasets analyzed in the present study are available from the published papers that have been cited in the present manuscript.
