# Supplementary material for: Efficacy and safety of glucocorticoids in the treatment of COVID-19: a systematic review and meta-analysis of RCTs
Source: Clin Exp Med. 2024 Jul 13;24(1):157. doi: 10.1007/s10238-024-01405-0 (PMC11246314; doi:10.1007/s10238-024-01405-0)

Supplemental file

Table 1. Search strategies

| 1 | MeSH terms | COVID-19, glucocorticoid, dexamethasone, prednisolone, prednisone |
| --- | --- | --- |
| 2 | Free-text terms | COVID 19, 2019-nCoV, 2019 nCoV, SARS-CoV-2, SARS CoV 2, 2019 Novel Coronavirus, Coronavirus disease 2019, COVID-19 Virus, COVID 19 Virus, SARS Coronavirus 2 Infection, Severe Acute Respiratory Syndrome Coronavirus 2 Infection, Decameth, Decaspray, Dexasone, Dexpak, Maxidex, Millicorten, Oradexon, Decaject, Decaject-L.A., Decaject L.A., Hexadrol, Dehydrocortisone, delta-Cortisone, Rectodelt, Prednison Hexal, Sterapred, Ultracorten, Winpred |
| 3 | Boolean framework | (((((((((((("COVID 19") OR ("2019-nCoV")) OR ("2019 nCoV")) OR (SARS-CoV-2)) OR ("SARS CoV 2")) OR ("2019 Novel Coronavirus")) OR ("Coronavirus disease 2019")) OR ("COVID-19 Virus")) OR ("COVID 19 Virus")) OR ("SARS Coronavirus 2 Infection")) OR ("Severe Acute Respiratory Syndrome Coronavirus 2 Infection")) OR (COVID-19[MeSH Terms])) AND ((((((glucocorticoids[MeSH Terms])) OR (dexamethasone[MeSH Terms])) OR (prednisolone[MeSH Terms])) OR (prednisone[MeSH Terms])) OR (((((((((((((((((((((glucocorticoid) OR (Methylfluorprednisolone)) OR (Hexadecadrol)) OR (Decameth)) OR (Decaspray)) OR (Dexasone)) OR (Dexpak)) OR (Maxidex)) OR (Millicorten)) OR (Oradexon)) OR (Decaject)) OR (Decaject-L.A.)) OR (Decaject L.A.)) OR (Hexadrol)) OR (Dehydrocortisone)) OR (delta-Cortisone)) OR (Rectodelt)) OR (Prednison Hexal)) OR (Sterapred)) OR (Ultracorten)) OR (Winpred)) |

Fig 1. The effect of corticosteroids on mortality at 28 days among moderate or severe ARDS COVID-19 patients.


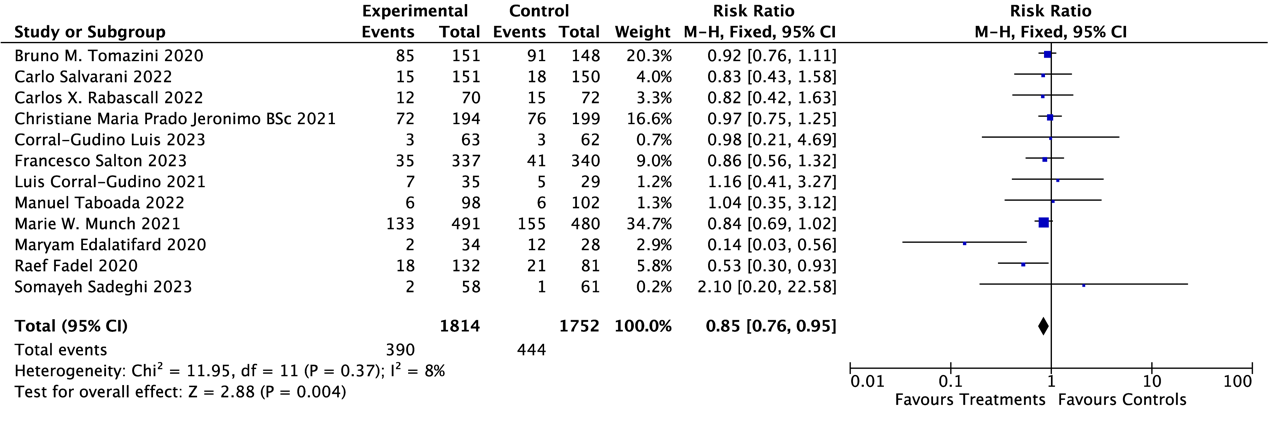


Fig.2 The effect of corticosteroids(Dexamethasone) on 28-VFD among moderate or severe ARDS COVID-19 patients.


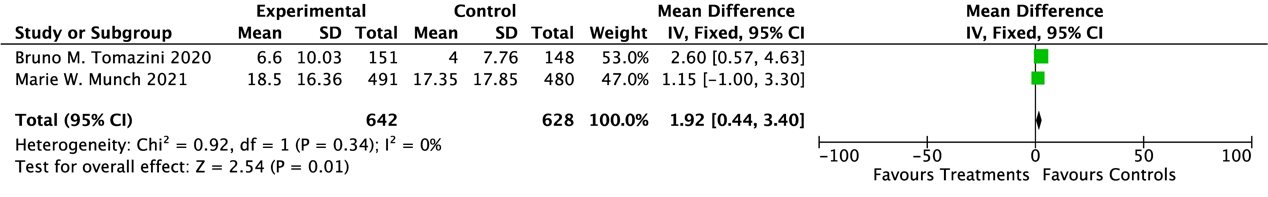


Fig.3 Subgroup Analysis of corticosteroid type on 28-day mortality rates among hospitalized patients.


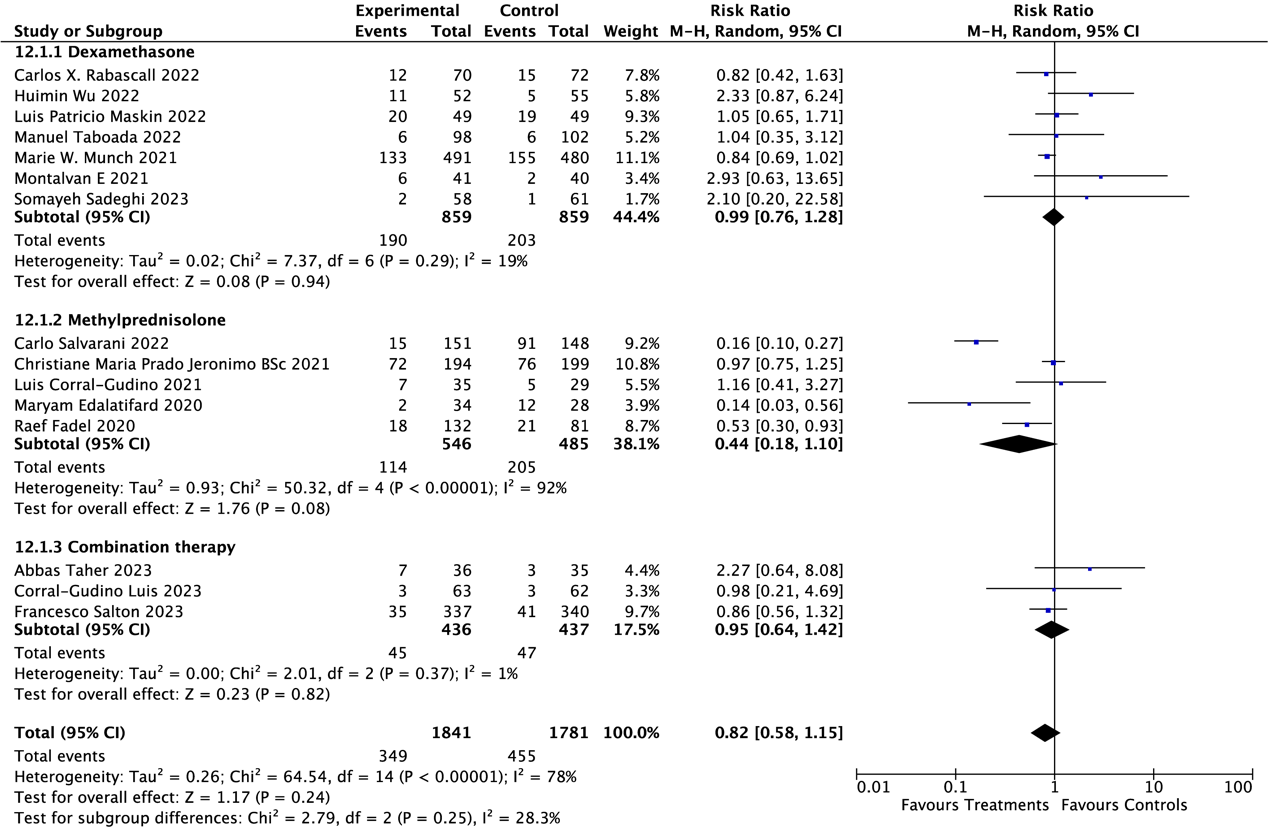


Fig.4 Subgroup Analysis of corticosteroid type on 28-day mortality rates among moderate or severe COVID-19 patients.


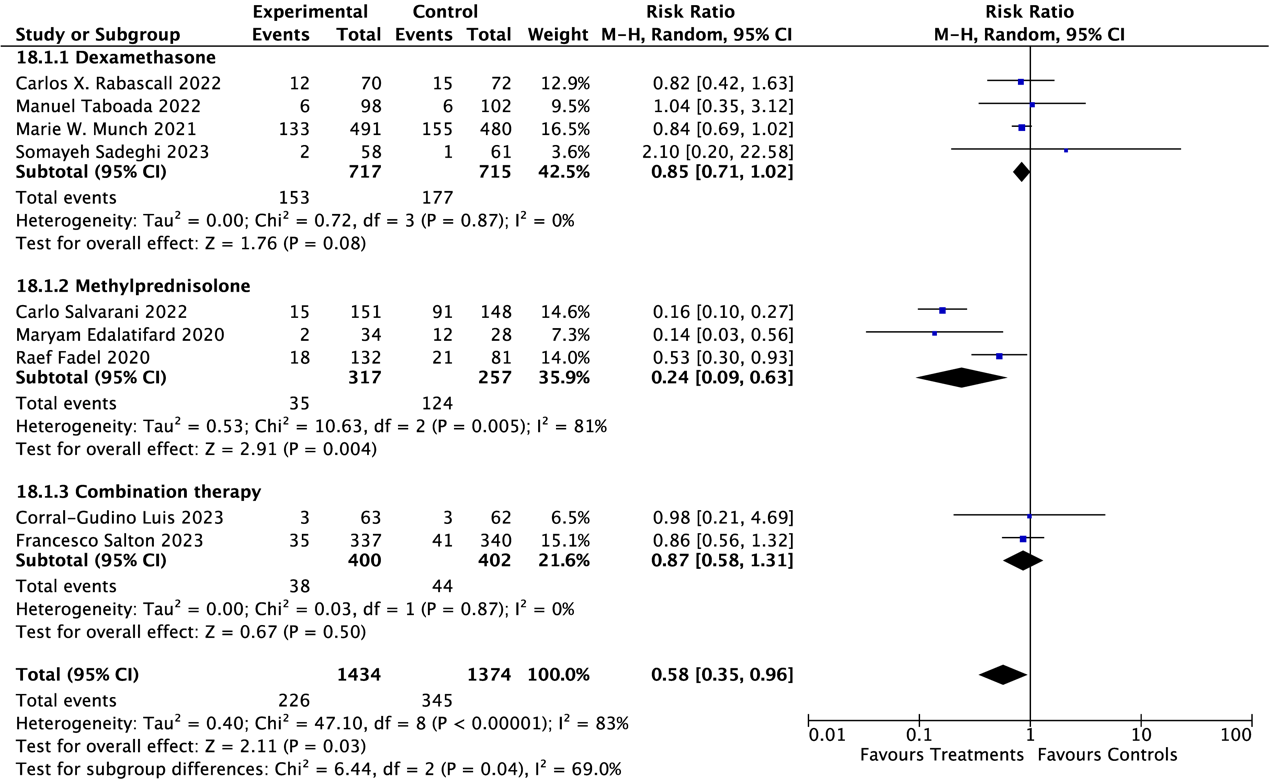


Fig.5 Subgroup Analysis of corticosteroid treatment duration on 28-day mortality rates among moderate or severe ARDS COVID-19 patients.


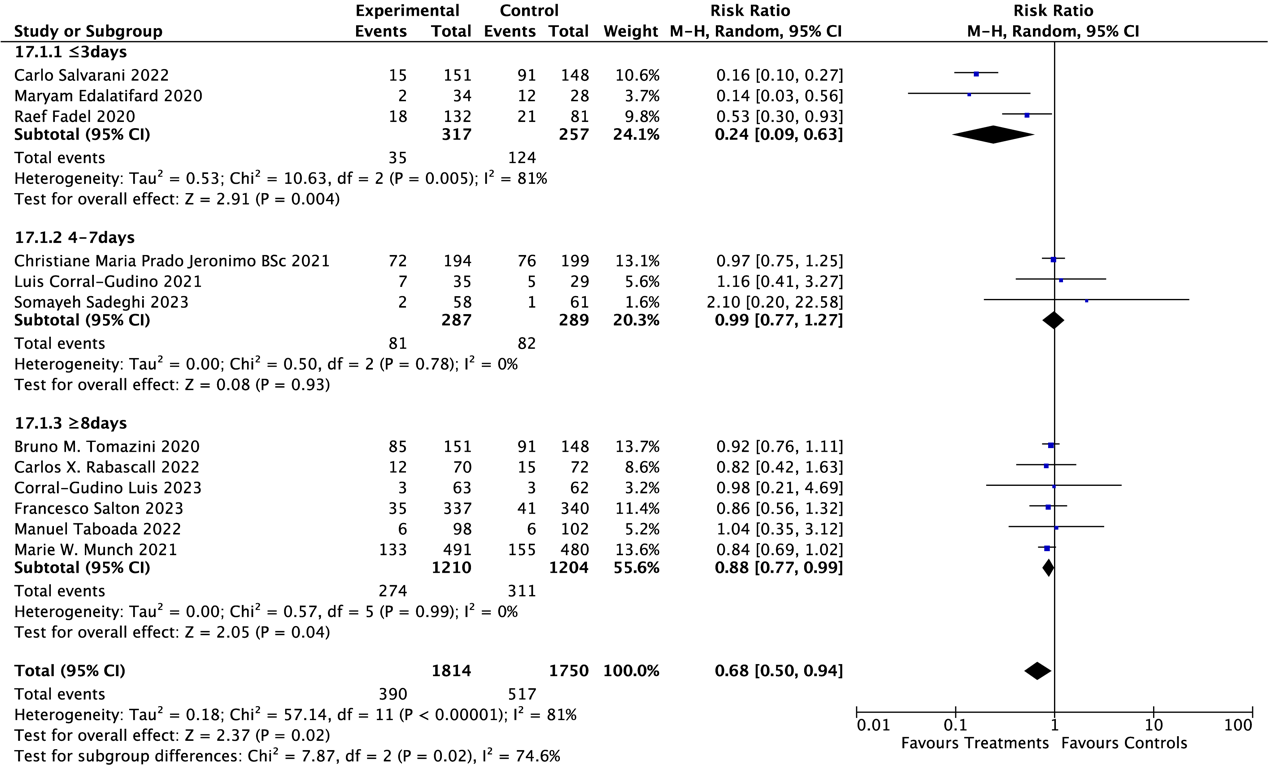

Supplement: Supplementary file 1 — Supplementary file1 (DOCX 11141 KB) [file 10238_2024_1405_MOESM1_ESM.docx]
